# Cold Shock Proteins: A Minireview with Special Emphasis on Csp-family of Enteropathogenic *Yersinia*

**DOI:** 10.3389/fmicb.2016.01151

**Published:** 2016-07-22

**Authors:** Riikka Keto-Timonen, Nina Hietala, Eveliina Palonen, Anna Hakakorpi, Miia Lindström, Hannu Korkeala

**Affiliations:** Department of Food Hygiene and Environmental Health, Faculty of Veterinary Medicine, University of HelsinkiHelsinki, Finland

**Keywords:** adaptation, Csp, cold stress, stress response, stress tolerance, *Yersinia enterocolitica*, *Yersinia pseudotuberculosis*

## Abstract

Bacteria have evolved a number of mechanisms for coping with stress and adapting to changing environmental conditions. Many bacteria produce small cold shock proteins (Csp) as a response to rapid temperature downshift (cold shock). During cold shock, the cell membrane fluidity and enzyme activity decrease, and the efficiency of transcription and translation is reduced due to stabilization of nucleic acid secondary structures. Moreover, protein folding is inefficient and ribosome function is hampered. Csps are thought to counteract these harmful effects by serving as nucleic acid chaperons that may prevent the formation of secondary structures in mRNA at low temperature and thus facilitate the initiation of translation. However, some Csps are non-cold inducible and they are reported to be involved in various cellular processes to promote normal growth and stress adaptation responses. Csps have been shown to contribute to osmotic, oxidative, starvation, pH and ethanol stress tolerance as well as to host cell invasion. Therefore, Csps seem to have a wider role in stress tolerance of bacteria than previously assumed. *Yersinia enterocolitica* and *Yersinia pseudotuberculosis* are enteropathogens that can spread through foodstuffs and cause an enteric infection called yersiniosis. Enteropathogenic *Yersinia* are psychrotrophs that are able to grow at temperatures close to 0°C and thus they set great challenges for the modern food industry. To be able to efficiently control psychrotrophic *Yersinia* during food production and storage, it is essential to understand the functions and roles of Csps in stress response of enteropathogenic *Yersinia*.

## Introduction

Bacteria encounter changing environmental conditions during food production and storage and they have evolved a number of mechanisms for coping with stress and adapting to changing environments. In modern food production refrigeration is one of the key elements in maintaining food safety. At cell level temperature downshift decreases the fluidity of cell membranes, which affects active transport and protein secretion (Phadtare and Severinov, 2010). In addition, the efficiency of transcription and translation is reduced due to stabilization of the secondary structures of DNA and RNA, protein folding is inefficient, and ribosomes need to be adapted to cold before they can function properly (Phadtare, 2004). The aim of cold shock response is to help bacterial cells to overcome these changes (Phadtare and Severinov, 2010).

Bacteria respond to a rapid temperature drop (cold shock) by a transient induction of cold induced proteins (Cips) (Graumann and Marahiel, 1996; Phadtare, 2004) and the production of Cips increases with the severity of the cold shock (Hébraud and Potier, 1999). In *Escherichia coli* numerous Cips have been identified so far including, e.g., cold shock protein (Csp) family (Yamanaka et al., 1998), RNA helicase csdA (Charollais et al., 2004), exoribonucleases PNPase and RNaseR (Phadtare, 2012), initiation factors 2α and 2β, NusA and RecA (Jones et al., 1987). This minireview focuses on a subgroup of Cips, the small homologous Csps that are classified together in the Csp-family.

Cold shock proteins counteract some harmful effects of temperature downshift and thus help the cells to adapt (Phadtare, 2004). After the immediate cold shock response, the synthesis of Csps declines and synthesis of other proteins increases. This enables the cells to grow at low temperature, although at a slower rate (Ermolenko and Makhatadze, 2002). Csps are known to be important during cold shock response, however, recent studies have shown that Csps might have a wider role in stress tolerance of bacteria (Schmid et al., 2009; Duval et al., 2010; Loepfe et al., 2010; Michaux et al., 2012; Schärer et al., 2013; Wang et al., 2014; Derman et al., 2015).

*Yersinia enterocolitica* and *Yersinia pseudotuberculosis* are enteropathogens that can cause a foodborne enteric infection called yersiniosis. In 2013, yersiniosis was the third most frequently reported zoonosis in the European Union (EFSA and ECDC, 2015). Food products containing pork are considered to be the main vehicles of *Y. enterocolitica* infection (Fredriksson-Ahomaa et al., 2006), whereas large *Y. pseudotuberculosis* outbreaks have been linked to contaminated vegetables (Nuorti et al., 2004; Jalava et al., 2006), drinking water (Fukushima et al., 1988), and raw milk (Pärn et al., 2015).

*Yersinia enterocolitica* and *Y. pseudotuberculosis* are psychrotrophic bacteria that can grow even at temperatures close to 0°C (Fredriksson-Ahomaa et al., 2010). The control of *Yersinia* in the modern food chain involving refrigeration as the sole means to increase the shelf life of food products is thus challenging. To be able to efficiently control enteropathogenic *Yersinia* in refrigerated foods, a thorough knowledge of their cellular adaptation mechanisms to changing environments during food production and storage is needed. This minireview summarizes the current understanding of the functions of Csp-family proteins and highlights what is currently known about their role in stress response of enteropathogenic *Yersinia*.

## Cold Shock Proteins

Cold shock proteins are small nucleic acid-binding proteins ranging from 65 to 75 amino acids in length (Graumann and Marahiel, 1996; Czapski and Trun, 2014) and they have been found in psychrophilic, mesophilic, thermophilic and even hyperthermophilic bacteria (Phadtare, 2004; Jin et al., 2014). CspA was first described in *E. coli* (Goldstein et al., 1990). Later it was found that *E. coli* CspA family consists of nine homologous proteins named CspA through CspI that share a 46–91% amino acid sequence similarity (Yamanaka et al., 1998). Similar naming has been used in other bacteria as well, but genes with identical names do not necessarily share identical structure or function in different bacteria.

Structurally Csps are highly conserved, however, their ther- mostability varies (Lee et al., 2013; Jin et al., 2014). CspA of the psychrotrophic *Listeria monocytogenes* has a melting temperature of 40°C (Lee et al., 2013), whereas Csp of thermophilic *Thermus aquaticus* has a more rigid structure and a melting temperature as high as 76°C, suggesting that psychrophilic Csps need higher structural flexibility to accommodate nucleic acids upon cold shock (Jin et al., 2014). At 37°C, the *cspA* mRNA of *E. coli* is very unstable, its half-life being only 12 s, but after cold shock its stability increases producing a half-life of more than 20 min (Mitta et al., 1997). Transient *cspA* mRNA stabilization due to low temperature is probably an important factor in the induction of CspA during cold shock (Phadtare and Severinov, 2005).

## Function of Cold Shock Proteins During Cold Shock

Csps have a highly conserved nucleic acid binding domain, called the cold shock domain (CSD) (Graumann and Marahiel, 1996). CSD contains two nucleic acid binding motifs, ribonucleoprotein 1 and 2 (Lee et al., 2013) that facilitate binding to target RNA and DNA (Chaikam and Karlson, 2010). Jiang et al. (1997) demonstrated that CspA of *E. coli* binds weakly and with low sequence specificity to ssRNA. Csps function as RNA chaperones by destabilizing secondary structures in target RNA at low temperature so that the single-stranded state of target RNA is maintained. This enables efficient transcription and translation (Jiang et al., 1997; Phadtare, 2004). The weak and low sequence specific binding of RNA is important for the chaperone function of CspA and its homologues. Otherwise the movement of ribosomes on target mRNA would be hindered (Yamanaka, 1999). Due to the chaperone function, Csps can also act as transcription antiterminators by preventing formation of hairpin structures, which can act as transcriptional termination or pause sites in target RNA at low temperatures (Bae et al., 2000; Phadtare et al., 2002).

The *cspA* mRNA of *E. coli* can sense temperature changes and adapt to these changes by adopting different functional structures (Giuliodori et al., 2010). Giuliodori et al. (2010) showed that at low temperature the *cspA* mRNA derived from *E. coli* undergoes a temperature dependent structural change and as a result the *cspA* mRNA is translated more efficiently and is less prone to degradation than the *cspA* mRNA structure at 37°C. Similarly, Mega et al. (2010) discovered that cold-induced *ttcsp2* of thermophilic *Thermus thermophilus* acts as a thermosensor by adopting a more stable secondary structure due to a temperature drop.

Only CspA, B, CspE, G and CspI of *E. coli* are induced by cold shock (Etchegaray et al., 1996; Nakashima et al., 1996; Wang et al., 1999; Uppal et al., 2008) (**Table 1**). Xia et al. (2001) discovered that in *E. coli* four out of nine *csp* genes (*cspA, cspB, cspE*, and *cspG*) had to be deleted until cold sensitive phenotype was obtained. In addition, deletion of one or two *csp* genes increased and prolonged the expression of the remaining cold induced *csp* genes. This indicates that the functions of the CspA family members overlap and they can compensate for each other (Xia et al., 2001). Similarly in *Bacillus subtilis*, the loss of one or two *csp* genes increased the production of remaining Csps after cold shock (Graumann et al., 1997). In *E. coli*, temperature fluctuation between 37°C and 8°C increased the *cspA* and *cspB* transcription during each temperature downshift whereas transcription decreased when temperature was raised (Ivancic et al., 2013). However, the CspA and CspB protein concentrations were only increased during the first temperature downshift and the relative protein levels remained constant during temperature fluctuations suggesting that the proteins are rather stable and not degraded at higher temperature (Ivancic et al., 2013). Csps may aid bacteria to survive in extremely cold polar environment. Jung et al. (2010) discovered that when the CspA of psychrophilic *Psychromonas arctica* isolated from Artic sea sediments was overexpressed in *E. coli*, the survival of cells after repeated freezing and thawing increased over 10-fold.

**Table 1:** Cold shock protein genes of *Escherichia coli*, their reported functions and the predicted *csp* genes of *Yersinia enterocolitica* 8081 and *Yersinia pseudotuberculosis* IP32953 that share the highest amino acid sequence similarity (in percentage)^a^ with *csp* genes of *Escherichia coli* K-12 W3110.

			*csp g*ene with highest similarity in *Y. enterocolitica* 8081		*csp g*ene with highest similarity in *Y. pseudotuberculosis* IP32953	
*csp* genes of* E. coli*	Reported function	Reference(s)	Gene name(s)	Amino acid sequence similarity (%)	Gene name(s)	Amino acid sequence similarity (%)
*cspA*	Induced by cold Major cold shock protein of *E. coli*	Jones et al., 1987; Goldstein et al., 1990	YE3823	78.57	YPTB3587	74.29
*cspB*	Induced by cold	Etchegaray et al., 1996	YE3823	80.00	YPTB3585, YPTB3586	77.14
*cspC*	Involved in regulation of expression of stress response proteins RpoS and UspA Involved in regulation of growth	Phadtare and Inouye, 2001; Rath and Jawali, 2006; Cohen-Or et al., 2010; Shenhar et al., 2012	YE2590, YE3012	81.16	YPTB1624	98.55
*cspD*	Induced by stationary phase growth and nutrient starvation Involved in persister cell formation and biofilm development Inhibits DNA replication Overproduction of CspD is toxic for the cell	Yamanaka and Inouye, 1997; Xia et al., 2001; Yamanaka et al., 2001; Kim and Wood, 2010; Kim et al., 2010	YE1516	83.78	YPTB1392	83.78
*cspE*	Induced by cold Involved in regulation of expression of stress response proteins RpoS and UspA CspE constitutively produced at 37°C, increase in production during lag phase	Bae et al., 1999; Phadtare and Inouye, 2001; Shenhar et al., 2012; Czapski and Trun, 2014	YE3012	91.30	YPTB1088	94.20
*cspF*	Expressed at very low level, no protein has been detected from the gene. Function unknown	Czapski and Trun, 2014	YE3821, YE3822, YE3823	48.57	YPTB3585, YPTB3586, YPTB3587	48.57
*cspG*	Induced by cold	Nakashima et al., 1996	YE3823	81.43	YPTB2950, YPTB3587	77.14
*cspH*	Expressed at very low level, no protein has been detected from the gene. Function unknown	Czapski and Trun, 2014	YE3821, YE3822, YE3823	50.00	YPTB3585, YPTB3586, YPTB3587	51.43
*cspI*	Induced by cold	Wang et al., 1999	YE3823	84.29	YPTB3587	82.86

*^a^Alignments were calculated using Clustal Omega v.1.2.1. Nucleotide and amino acid sequences were derived from The Kyoto Encyclopedia of Genes and Genomes (Kanehisa et al., 2016).*

Mesophilic *Clostridium botulinum* Group I and III strains have two or three *csp* genes (*cspA, cspB* and *cspC*), but of the psychrotrophic *Clostridium botulinum* Group II strains, only one type B toxic strain carries one *csp* gene, whereas none of type E toxic strains has *csp* genes (Söderholm et al., 2013). The lack of *csp* genes in psychrotrophic Group II *C. botulinum* indicates that the cold tolerance of these strains is due to some other mechanism. In *C. botulinum* ATCC3502 inactivation of just one of the three *csp* genes, *cspB*, resulted in cold sensitive phenotype, suggesting that *cspB* is the major Csp of Group I *C. botulinum* (Söderholm et al., 2011). This is also supported by the fact that all *C. botulinum* Group I and III strains have homologues for *cspB* (Söderholm et al., 2013).

## Cold Shock Proteins of Enteropathogenic *Yersinia*

The published genome sequences of enteropathogenic *Yersinia* reveal that they carry several putative Csp encoding genes. *Y. enterocolitica* and *Y. pseudotuberculosis* strains have 6–10, and 7–9 *csp* genes, respectively. The Csps of *Yersinia* are highly homologous, and the cold-shock proteins encoding genes of, e.g., *Y. pseudotuberculosis* IP32953 share 32.8–100% amino acid and 44.6–99.5% nucleotide sequence similarity (**Table 2**). When the *csp* genes of *E. coli* K-12 W3110 are compared to the most closely similar *csp* genes of *Y. enterocolitica* 8081 and *Y. pseudotuberculosis* IP32953, the amino acid sequence similarity varies between 48.6 and 98.6% (**Table 1**). Since some of the Csp encoding genes of enteropathogenic *Yersinia* share a very high amino acid sequence similarity (74.3–98.6%) with *cspA, cspB, cspC, cspD, cspE, cspG, and cspI* of *E. coli* it can be assumed that they also have common functions. Lower amino acid sequence similarity is observed with *E. coli* genes *cspF* and *cspH* which function in *E. coli* is unknown (Czapski and Trun, 2014).

**Table 2:** Nucleotide and amino acid sequence similarity (in percentage)^a^ of the putative cold shock proteins of *Yersinia pseudotuberculosis* strain IP32953.

Gene name^b^	yptb1088	yptb1392	yptb1423	yptb1624	yptb2414	yptb2950	yptb3585	yptb3586	yptb3587
yptb1088 (210 bp)	—	46.27	53.62	84.06	79.71	66.67	73.91	73.91	72.46
yptb1392 (264 bp)	57.84	—	32.84	46.27	47.76	49.25	46.27	46.27	47.76
yptb1423 (210 bp)	60.00	44.61	—	49.28	49.28	47.83	49.28	49.28	47.83
yptb1624 (210 bp)	75.24	53.92	58.10	—	82.61	75.36	73.91	73.91	72.46
yptb2414 (213 bp)	71.43	52.94	57.62	84.76	—	74.29	78.57	78.57	77.14
yptb2950 (213 bp)	64.76	56.37	51.90	72.86	70.42	—	92.86	92.86	88.57
yptb3585 (213 bp)	66.67	56.37	54.76	73.33	80.75	84.04	—	100.00	95.71
yptb3586 (213 bp)	67.14	56.37	55.24	72.86	80.28	83.57	99.53	—	95.71
yptb3587 (213 bp)	66.67	57.35	53.81	72.86	79.34	83.10	95.77	96.24	—

*^a^Translated amino acid sequence comparisons above the diagonal and nucleotide sequence comparisons below the diagonal. Alignments were calculated using Clustal Omega v.1.2.1. Nucleotide and amino acid sequences were derived from The Kyoto Encyclopedia of Genes and Genomes (Kanehisa et al., 2016). ^b^Gene length in parentheses.*

*Yersinia enterocolitica, Y. pseudotuberculosis, Yersinia pestis*, and *Yersinia ruckeri* carry a locus containing a tandem *cspA* duplication (*cspA1* and *cspA2*) which produces both monocistronic (CspA1) and bicistronic (CspA1/A2) mRNA templates (Neuhaus et al., 1999). At high temperatures monocistronic mRNA predominates. When the temperature decreases, more bicistronic mRNA is produced but the longer the cold shock lasts, the more monocistronic mRNA is produced (Neuhaus et al., 1999). Compared to *E. coli*, these mono- and bicistronic mRNA templates give *Y. enterocolitica* a better transcriptional capacity during the cold shock. Synthesis of proteins is more efficient when the transcript contains two copies of the protein (Neuhaus et al., 1999).

Annamalai and Venkitanarayanan (2005) studied the Csp expression of *Y. enterocolitica* in Luria-Bertani (LB) broth, milk, and on pork meat after temperature drop from 30°C to 4°C. CspA1 and CspA2 were first detected 2 h after cold shock from LB and milk cultures, but it took as long as 8 h after cold shock to observe detectable levels of Csps on pork meat. On pork meat the delayed expression of Csps and genes might be due to the extended lag phase of *Y. enterocolitica* on solid meat surface compared to liquid medium. Both in LB broth and on pork meat the expression of Csps and genes continued until 24 h of cold shock suggesting that in *Y. enterocolitica* Csps are not only needed during the immediate cold shock response but also during the cold acclimation. At 30°C, CspA1 and CspA2 were not observed at all (Annamalai and Venkitanarayanan, 2005).

After cold adaptation, Csp mRNAs must be degraded to liberate ribosomes for translation of mRNAs of non-Csps and thereby to enable growth to resume at low temperature (Neuhaus et al., 2000). Neuhaus et al. (2000) observed correlation between beginning of exponential growth of *Y. enterocolitica* cells and degradation of *cspA1/A2* mRNA. After adaptation to cold, *Y. enterocolitica cspA1/A2* transcripts were smaller than the original transcript, and all cut off at the same sequence (AGUAAA) that was later named the cold shock cut box (CSC-box) (Neuhaus et al., 2003). Mutation of the CSC-box caused a delayed growth resume after cold shock. The CSC-box facilitates the degradation of Csp mRNA. Endonuclease RNaseE first cuts the *cspA1/A2* transcript to smaller pieces which are further degraded by PNPase (Neuhaus et al., 2003). PNPase is necessary for *Y. enterocolitica* growth at low temperature (Goverde et al., 1998) and it regulates gene expression by selectively degrading Csp mRNAs (Yamanaka and Inouye, 2001b).

## Role of Cold Shock Proteins During Normal Growth and in Stress Responses Unrelated to Cold

Cold shock proteins are not only produced during cold stress. A minimum of one *csp* gene is essential for viability of *B. subtilis*, suggesting that Csps are needed during non-shock growth (Graumann et al., 1997). In *E. coli* CspA forms 1% of all soluble proteins at the early exponential growth phase at 37°C, suggesting that CspA has functions also at optimal growth temperature (Brandi et al., 1999). In *E. coli*, both growth medium and growth phase have been shown to affect the transcription of *csp* genes (Czapski and Trun, 2014). In MOPS defined minimal glucose medium *cspE* mRNA levels were higher than mRNA levels of other *csp* genes, whereas in MOPS defined rich glucose medium transcripts for *cspA, cspB, and cspE* predominated (Czapski and Trun, 2014). In *E. coli* transcripts of *cspA* predominate during the lag phase and first stages of logarithmic growth whereas only small levels of *cspA* mRNA can be detected at other stages of growth (Brandi and Pon, 2012). Also nutritional upshift has been shown to induce CspA in *E. coli*, although the related level of induction was only one-sixth of that caused by cold shock (Yamanaka and Inouye, 2001a).

Functions of non-cold-inducible Csps are yet poorly understood (Tanaka et al., 2012). DNA microarray analysis revealed that deletion of *ttcsp1*, which is the only non-cold inducible *csp* gene of *T. thermophilus*, did not alter the gene expression profile compared to wild type at optimal growth conditions. Nevertheless, expression levels of some proteins were significantly upregulated or downregulated in *Δttcsp1*, suggesting that ttCsp1 contributes to translational control (Tanaka et al., 2012). Tanaka et al. (2012) also suggested that different stress factors might alter the nucleotide binding affinities of some Csps. On the contrary, the whole transcriptome of a *cspA* mutant of *Brucella melitensis* revealed that a total of 446 genes were differentially expressed compared to wild type. Differences in expression were especially observed in genes associated with virulence and metabolism and the *cspA* mutant also showed reduced growth in minimal medium. Results suggest that *cspA* of *B. melitensis* plays a role in virulence and metabolism regulation, but the CspA-mediated regulatory mechanisms are not understood (Wang et al., 2016). These contrary results observed in gene expression of different *csp* mutants warrant that further research is needed to understand by which mechanism Csps function during normal growth and in stress responses unrelated to cold.

In *E. coli, cspD* inhibits DNA replication and is induced during stationary phase to resign growth. Overproduction of this Csp is thus toxic to the cells (Yamanaka et al., 2001; Uppal et al., 2014). CspD has also been linked to biofilms and persister cell formation (Kim and Wood, 2010; Kim et al., 2010). Recently it was also shown that Csps might have a role in buffering deleterious mutations since overexpression of CspA improved fitness of *E. coli* strains that had accumulated deleterious mutations during long-term laboratory experiments (Rudan et al., 2015). Rudan et al. (2015) suggested that CspA and other RNA chaperones could aid misfolded RNA to adopt functional conformation and thus suppress harmful mutations that affect RNA structure. Since mutation rate can be elevated in response to stress (Hersh et al., 2004; Foster, 2007), it would of interest to investigate whether Csps could also counteract harmful stress-induced mutations.

Derman et al. (2015) revealed that *cspB* and *cspC*, but not *cspA*, play an important role in NaCl, pH and ethanol stress in *C. botulinum* ATCC3502. Mutation of *cspA* and *cspC* also reduced motility and hampered flagella formation. In *L. monocytogenes* CspA is the major cold-shock-responsive Csp, whereas CspB and CspD are involved in host cell invasion (Schmid et al., 2009; Loepfe et al., 2010). Deletion of one or both of *cspB* and *cspD* resulted in severely impaired Caco-2 cell invasion, whereas deletion of *cspA* had no effect on cell invasion. Simultaneous deletion of *cspB* and *cspD* or deletion of all three *csp* genes of *L. monocytogenes* resulted in most severe impairment of host cell invasion and also caused reduced intracellular growth in macrophages (Loepfe et al., 2010). Schärer et al. (2013) suggested that Csps contribute to regulation of listeriolysin O, which is a pore-forming cytolysin needed in intracellular survival. In addition, *cspA* contributed to oxidative stress tolerance (Loepfe et al., 2010), and single deletion of *cspD*, but not of *cspA* and *cspB*, resulted in impaired osmotic stress adaptation in *L. monocytogenes* (Schmid et al., 2009). Since Csps promote *L. monocotygenes* adaptation against different stress conditions, exposure to one type of stress, e.g., during food production might induce cross-protection against others (Schmid et al., 2009).

## Conclusion

Production of Csps is one of the most prominent responses of bacteria to cold shock and recent studies have shown that Csps have a much wider role in stress response of bacteria than previously assumed. Enteropathogenic *Yersinia* encode several Csps which show a high homology to those of *E. coli*. Some of these *E. coli csp* genes are linked to stresses other than cold. *Y. enterocolitica* CspA1 and CspA2 are known to be involved in cold shock response, however, it is not known what the role of other *csp* genes is in stress response of enteropathogenic *Yersinia.* Therefore, knowledge about the function of Csps is needed to be able to develop measures that limit the growth and survival of enteropathogenic *Yersinia* in various foods during food production and storage.

## Author Contributions

RK-T, NH, EP, ML, and HK designed the minireview, RK-T and AH performed sequence comparisons, RK-T and NH drafted the manuscript, EP, ML, and HK critically revised the manuscript.

## Conflict of Interest Statement

The authors declare that the research was conducted in the absence of any commercial or financial relationships that could be construed as a potential conflict of interest.
